# Home delivery among pregnant women with ANC follow-up in Ethiopia; Evidence from the 2019 Ethiopia mini demographic and health survey

**DOI:** 10.3389/fpubh.2022.862616

**Published:** 2022-11-16

**Authors:** Mandaras Tariku, Daniel Berhanie Enyew, Biruk Shalmeno Tusa, Adisu Birhanu Weldesenbet, Nebiyu Bahiru

**Affiliations:** ^1^Department of Psychiatry, College of Health and Medical Sciences, Haramaya University, Harar, Ethiopia; ^2^Department of Public Health and Health Policy, School of Public Health, College of Health and Medical Sciences, Haramaya University, Harar, Ethiopia; ^3^Department of Epidemiology and Biostatistics, School of Public Health, College of Health and Medical Sciences, Haramaya University, Harar, Ethiopia

**Keywords:** ANC, home delivery, pregnancy, Ethiopia, antenatal care

## Abstract

**Background:**

Maternal mortality has remained an international public health problem although it is decreasing in recent years. Developing countries particularly Sub-Saharan African countries bears the high burden of maternal deaths. There was no study conducted to assess prevalence and associated factors of home delivery among women in Ethiopia on antenatal care (ANC) follow up nationally. Therefore, this study was conducted to assess the magnitude and associated factors of home delivery in Ethiopia.

**Objectives:**

To assess the magnitude of home delivery and associated factors among women who had ANC follow up in Ethiopia.

**Methods:**

Secondary data analysis was carried out using Ethiopian Mini Demography and Health Survey (EMDHS 2019). A total weighted sample of 2,143 women who had ANC follow up during pregnancy was incorporated in the study. In a generalized linear mixed model (GLMM), Adjusted Odds Ratio (AOR) with a 95% Confidence Interval (CI) and *p* < 0.05 were declared as associated factors of home delivery.

**Results:**

The prevalence of home delivery was 31.27% [95% CI: 29.34%, 33.27%] among women who had ANC follow up in Ethiopia. Attended higher education [AOR = 0.27; 95% CI: (0.13, 0.54)], rural resident [AOR = 2.15; 95% CI: (1.19, 3.90)], richest in the wealth index [AOR = 0.18; 95% CI: (0.10, 0.32)], had adequate ANC follow up [AOR = 0.25; 95% CI: (0.13, 0.51)] and being in third trimesters [AOR = 0.64; 95% CI: (0.49, 0.83)] during first ANC visit were significantly associated factors of home delivery.

**Conclusion:**

Near to one-third of women in Ethiopia have delivered their babies at home even if they had an ANC follow up. Educational status, place of residence, wealth index, timing of first antenatal check and adequate ANC visit has shown significant association with home delivery. Therefore, focused intervention packages need to be implemented at all levels of the health care system in Ethiopia to improve health seeking behaviors of women who have ANC follow up to have delivery in health care institutions. While doing so, special attention should be given for poor, uneducated and rural dweller women.

## Background

Home delivery is delivering a baby in a non-clinical and unsanitary residence (1, 2). It makes it difficult to tackle catastrophic complications of child birth like postpartum hemorrhage, sepsis; pregnancy-induced hypertension, and obstructed labor which are the major causes of maternal mortality worldwide. Most of deliveries in developing counties are responsible for the majority of maternal deaths. Delivery by skilled birth attendant is critical in saving women lives (3–5).

Although there is a decrease in the past years, maternal mortality has remained an international public health challenge. Globally, maternal death declined from 342 to 211 per 100,000 live births between 2000 and 2017 (4). There is a very wide gap between the developed and developing nations in terms of risks of women dying from pregnancy, labor and delivery-related complications (1, 6). The highest maternal death of 640 women per 100,000 live births were reported from sub-Saharan Africa, followed by South Asia (280 deaths per 100,000 live births) in 2015 (7). Moreover, one in 22 women dies of obstetric complication in Sub-Saharan Africa (8, 9).

In the Ethiopian context though there has been a decreasing pattern of maternal mortality over the last 15 years, there is still an unacceptably high prevalence of maternal death (10). The Maternal mortality ratio of the country is 412 per 100,000 live births. Evidence illustrates that 50% of maternal mortality in developing countries including Ethiopia is attributed to unsafe delivery practices. The prevalence of home delivery in Ethiopia among women on ANC follow up ranges from 13.5% (11) reported in 2017 to 66.7% reported in 2020 (1).

The Ethiopian government drafted and successfully implemented a nationwide health transformation sector plan, which includes increasing the number of health care professionals. Furthermore, creating a women's development army community-level structure that will facilitate ANC and skilled delivery was paramount. In addition, it has been building health centers and primary hospitals that give basic and comprehensive maternal and neonatal health services for the rural and hard-to-reach areas over the last 15 years (6).

Antenatal Care describe the medical procedures and care that are carried out during pregnancy and currently a minimum of eight visit is recommended by WHO (12). Women who attended ANC would have a better understanding of the benefits of institutional delivery and make birth preparations ahead of time to deliver in a health facility. However, there is paucity of evidence on the prevalence and associated factors of home delivery among women who have ANC follow-up in Ethiopia nationally. Few studies conducted on the topic used small sample size and assessed home delivery in small geographic areas (11, 13).

Therefore, this study was conducted to assess the prevalence and associated factors of home delivery in Ethiopia among women who have ANC follow-up using the 2019 EDHS. The finding of this study helps to provide input for health planners and policymakers in designing specific interventions to increase facility level delivery.

## Methods

### Study setting and data source

Secondary data analysis was carried out using the Ethiopian Mini Demography and Health Survey (EMDHS 2019). The 2019 EMDHS was the second Mini Demographic and Health Survey conducted among from March 21, 2019, to June 28, 2019. Detailed information was collected on respondents' background characteristics, fertility determinants, family planning methods, maternal health care, child feeding practices, nutritional status of children, childhood vaccinations, and childhood mortality. This report presents full survey results for Ethiopia's nine regional states, two city administrations, and the nation as a whole.

Ethiopia is found in Eastern Africa has nine regions (Afar, Tigray, Amhara, Oromia, Somali, Southern Nations, Nationalities, and People's Region (SNNPR), Benishangul Gumuz, Gambella and Harari) and two administrative cities (Addis Ababa and Dire Dawa). Data were taken from the DHS measure website after getting permission.

### Sampling procedure, study population and sample size

The two stages of stratified sampling technique were applied in the EMDHS to select the study participants. Randomly, the Enumeration Areas (EA) was nominated in the first stage and then households were chosen in the second stage. The detail sampling procedure is available in the full 2019 EMDHS report (14).

For this study we used individual recording (IR) file and in this data set there were 8,885 records for all births. From this, we took 3,979 record for last birth. Then we included a total weighted sample of 2,143 women who were attended for ANC for their last birth for the final analysis. The detail data extraction procedure were followed according to Guide to DHS Statistics (15).

### Study variables

Place of delivery was considered as the dependent variable in this study. If the delivery took place at a respondent's home or others' home, it was coded as “Yes = 1.” In contrast, if the place of delivery was in health institutions (including government hospitals, health centers, health posts, private clinics, or private hospitals), it was coded as “No = 0.” Age, religion, marital status, educational level, place of residence, region, wealth index, sex of head of household, age of head of household, timing of 1st antenatal check, adequate ANC visits (if a woman has at least 4 ANC visits), getting laboratory investigation were included as independent variables in this study.

### Data processing and statistical analysis

Data was extracted, cleaned, recoded and analyzed using STATA software version 16.0. The data was weighted using sampling weight (women's sample weight), primary sampling unit, and strata. Descriptive statistics and summary statistics were presented in form of text, figure and tables. The prevalence of home delivery was presented using a pie chart.

Ethiopia mini demographic and health survey (EMDHS) data have cluster (village) nature and because of this women within a cluster may be more similar to each other than women in other clusters. This may violate the assumption of independence of observations and equal variance across clusters. To handle this, a generalized linear mixed model (GLMM) was fitted to find associated factors of home delivery. The intra-cluster correlation coefficient (ICC) was calculated to measure the variation between clusters (the proportion of the total observed difference in home delivery attributable to cluster variations) and it was found to be > 0.10. Variables with *p* ≤ 0.2 in the bi-variable analysis were fitted in the multivariable model to measure the effect of each variable after adjusting for the effect of other variables. Adjusted Odds Ratio (AOR) with a 95% Confidence Interval (CI) and *p* < 0.05 in the multivariable model was declared as associated factors of home delivery.

## Results

### Background characteristics of respondents

In 2019 EDHS 0.3% of women didn't know about the total number of ANC visits they attended or had missing data and they were excluded from analysis. A total of 2,143 women who had ANC follow up were included from nine regional states and two city administrations. As shown in Table 1, 678 (31.66%) of them were in the age range of 25–29 years. The mean age of study participant was 28.21 (±6.31) years. The majority of the women (92.86%) were married at the time of the survey and 39.29% were uneducated. Concerning wealth index, 31.59% of the women were from households with richest wealth quintiles. Nearly two-thirds (65.98%) and near to one-third (32.43%) of the women were from rural areas and Oromia region respectively. Regarding ANC information, half of the women (50.06%) had first ANC visit in the second trimester and 60.88% had adequate ANC visits. Among those who had ANC visits, the majority (96.38%) got laboratory investigation.

**Table 1:** Background characteristics of women who have ANC follow up in Ethiopia, 2019 EMEDHS, (*N* = 2,143).

**Variables**	**Weighted frequency**	**Percent**
**Age**		
15–19	120	5.61
20–24	479	22.37
25–29	678	31.66
30–34	444	20.72
35–39	288	13.42
40–44	108	5.02
45–49	26	1.19
**Religion**		
Orthodox	946	44.12
Muslim	656	30.62
Protestant	527	24.60
Other^@^	14	0.66
**Marital status**		
Never married	24	1.10
Currently married	1,990	92.86
Formerly/ever married	129	6.04
**Educational level**		
Uneducated	842	39.29
Primary	886	41.35
Secondary	277	12.94
Higher	138	6.42
**Place of residence**		
Urban	729	34.02
Rural	1,414	65.98
**Region**		
Tigray	213	9.93
Afar	28	1.31
Amhara	555	25.91
Oromia	695	32.43
Somali	58	2.73
Benishangul	26	1.23
SNNPR	415	19.35
Gambella	15	0.70
Harari	8	0.39
Addis Ababa	115	5.36
Dire Dawa	14	0.66
**Wealth index**		
Poorest	253	11.80
Poorer	378	17.64
Middle	409	19.09
Richer	426	19.87
Richest	677	31.59
**Sex of head of household**		
Male	1,824	85.12
Female	319	14.88
**Age of head of household**		
15–19	9	0.42
20–24	98	4.58
25–29	400	18.68
30–34	418	19.51
35–39	422	19.68
40–44	316	14.75
45–49	176	8.20
>49	304	14.19
**Timing of 1st** **antenatal check**		
First trimester	908	42.39
Second trimester	1,073	50.06
Third trimester	162	7.56
**Adequate ANC visits**		
No	838	39.12
Yes	1,305	60.88
**Got laboratory investigation**		
No	78	3.62
Yes	2,065	96.38

^@^Catholic and traditional.

### Prevalence of home delivery

The prevalence of home delivery was 31.27 % [95% CI: 29.34%, 33.27%] among women in Ethiopia who have ANC follow up.

### Factors associated with home delivery

Before running multivariable analysis bi-variable analysis was conducted to identify significant variables at *p*-value of 0.2. Accordingly age, religion, marital status, educational status, region, wealth index, sex of household head, timing of first antenatal check, age of household head, having got laboratory investigation during first antenatal check and number of living children were significantly associated with home delivery and included in multivariable regression analysis.

In multivariable mixed effect GLM, religion, educational status, place of residence, region, wealth index, timing of first antenatal check and adequate ANC visit were significantly associated with home delivery. Religion was significantly associated with home delivery among women who had ANC follow up. Women who were Muslim had 46% less chance of delivering at home (AOR = 0.54, 95% CI 0.33 to 0.89) compared to women who were Orthodox Christians. For women from rural areas, the odds of home delivery was 2.15 times higher (AOR = 2.15, 95% CI 1.19 to 3.90) compared to women from urban areas.

Level of education was strongly and significantly associated with home delivery among women who had ANC follow up. Women who attained primary, secondary and higher educational levels were 44% (AOR = 0.56, 95% CI 0.42 to 0.75), 67% (AOR = 0.33, 95% CI 0.20 to 0.54) and 73% (AOR = 0.27, 95% CI 0.13 to 0.54) less likely to deliver at home respectively compared to women who were uneducated. Similarly, women from households with poorer, middle, richer and richest wealth quintiles were 45% (AOR = 0.55, 95% CI 0.37 to 0.82), 56% (AOR = 0.44, 95% CI 0.29 to 0.67), 76% (AOR = 0.24, 95% CI 0.15 to 0.38) and 82% (AOR = 0.18, 95% CI 0.10 to 0.32) less likely to deliver at home respectively compared to women from households with poorest wealth quintiles.

Region was another variable which showed significant association with home delivery among women who had ANC visits. The odds of delivering at home was lower by 68, 56, 69, and 68% among women from Benishangul (AOR = 0.32, 95% CI 0.14 to 0.73), SNNPR (AOR = 0.44, 95% CI 0.21 to 0.93), Addis Ababa (AOR = 0.31, 95% CI 0.10 to 0.96) and Dire Dawa (AOR = 0.32, 95% CI 0.12 to 0.85), respectively compared to women from Oromia region.

Timing of first antenatal check was also significantly associated with home delivery. Women who had first ANC visit during the third trimester had 29% lower odds of delivering at home (AOR = 0.81, 95% CI 0.78 to 0.85) compared to those who had first ANC visit during the first trimester of pregnancy. Similarly, the odds of delivering at home was 36% lower among women who had adequate ANC visits (AOR = 0.64, 95% CI 0.49, 0.83) compared to women without adequate ANC visits (Table 2).

**Table 2:** Bivariable and multivariable mixed effect GLM analysis of home delivery among women who have ANC follow in Ethiopia, 2021.

**Variables**	**Home delivery**		**Odds ratio [95% CI]**		***P*-value**
	**Yes**	**No**	**COR**	**AOR**	
**Age in years**					
15–29	367	912	1	1	
30–49	304	560	1.40 [1.10, 1.78]	0.80 [0.57, 1.12]	0.190
**Religion**					
Orthodox	279	666	1	1	
Muslim	170	486	1.19 [0.77, 1.84]	0.54 [0.33, 0.89]	0.015*
Protestant	210	317	1.62 [0.99, 2.66]	1.44 [0.84, 2.44]	0.181
Other^@^	10	4	3.32 [1.14, 9.64]	2.02 [0.70, 5.78]	0.191
**Marital status**					
Currently married	626	1,364	1	1	1
Never married	2	22	0.49 [0.17, 1.36]	048 [0.17, 1.37]	0.169
Formerly married	42	87	1.10 [0.71, 1.73]	0.88 [0.53, 1.45]	0.604
**Educational level**					
Uneducated	359	483	1	1	
Primary	260	626	0.42 [0.32, 0.56]	0.56 [0.42, 0.75]	<0.001*
Secondary	37	241	0.19 [0.12, 0.30]	0.33 [0.20, 0.54]	<0.001*
Higher	14	123	0.11 [0.06, 0.22]	0.27 [0.13, 0.54]	<0.001*
**Place of residence**					
Urban	142	587	1	1	
Rural	886	528	11.80 [6.87, 20.27]	2.15 [1.19, 3.90]	0.012*
**Region**					
Oromia	250	445	1	1	
Tigray	45	168	0.49 [0.19, 0.27]	0.47 [0.21, 1.09]	0.079
Afar	13	14	3.07 [1.17, 8.05]	2.01 [0.87, 4.64]	0.100
Amhara	182	373	0.92 [0.38, 2.21]	0.69 [0.32, 1.47]	0.337
Somali	28	31	2.82 [0.91, 8.69]	1.27 [0.48, 3.41]	0.629
Benishangul	4	22	0.36 [0.13, 0.97]	0.32 [0.14, 0.73]	0.007*
SNNPR	134	280	0.82 [0.33, 2.01]	0.44 [0.21, 0.93]	0.031*
Gambella	5	10	0.31 [0.51, 3.35]	0.90 [0.42, 1.94]	0.795
Harari	2	7	0.27 [0.10, 0.74]	0.96 [0.41, 2.23]	0.928
Addis Ababa	4	111	0.18 [0.15, 0.21]	0.31 [0.10, 0.96]	0.042*
Dire Dawa	1	13	0.12 [0.04, 0.35]	0.32[0.12, 0.85]	0.022*
**Wealth index**					
Poorest	155	98	1	1	
Poorer	171	207	0.44 [0.30, 0.64]	0.55 [0.37, 0.82]	0.003*
Middle	163	246	0.34 [0.23, 0.50]	0.44 [0.29, 0.67]	<0.001*
Richer	100	326	0.15 [0.09, 0.22]	0.24 [0.15, 0.38]	<0.001*
Richest	82	595	0.05 [0.03, 0.08]	0.18 [0.10, 0.32]	<0.001*
**Sex of head of household**					
Male	582	1,242	1	1	
Female	88	231	1.01 [0.73, 1.38]	1.10 [0.76, 1.58]	0.618
**Age of head of household**					
15–29	120	387	1	1	
30–44	374	782	1.33 [1.00, 1.79]	1.11 [0.79, 1.55]	0.537
>44	176	303	1.45 [1.03, 2.05]	1.12 [0.84, 1.50]	0.741
**Timing of 1st antenatal check**					
First trimester	223	686	1	1	
Second trimester	351	721	1.49 [1.15, 1.92]	1.06 [0.81, 1.38]	0.675
Third trimester	96	66	5.05 [3.09, 8.25]	0.81 [0.78, 0.85]	0.001*
**Adequate ANC visits**					
No	346	492	1	1	
Yes	324	981	0.45 [0.35, 0.57]	0.64 [0.49, 0.83]	0.001*
**Got laboratory investigation during 1**^**st**^ **antenatal check**					
No	44	34	1	1	
Yes	626	1,439	0.37 [0.19, 0.72]	0.63 [0.32, 1.21]	0.166
**Number of living children (mean** **±SD)**	3.64 [±2.09]	2.79 [±2.00]	1.16 [1.10, 1.23]	1.06 [0.98, 1.15]	0.123
ICC (%)				24.23	

*P < 0.05; AOR, adjusted odds ratio; COR, crud odds ratio; SD, standard deviation; ICC, Intra-class correlation coefficient.

## Discussion

Even though the number of pregnant mothers in Ethiopia who attend antenatal clinics in healthcare facilities is increasing, skilled delivery service has remained very low. Individual level or health system-related factors contribute to women's preferences for delivery places. The predominant factors associated with home delivery services were lack of knowledge about obstetrics care, delay in starting ANC follow up, illiteracy and rural residence (16–19).

In this study, the weighted prevalence of home delivery among women who had ANC follow up was 31.27%. The prevalence is lower than the study conducted in Delanta District, South Wollo zone, North East Ethiopia, which is 35.2% (20). The magnitude difference between the two studies might be due to the fact that this study was conducted from the secondary data of EMDHS report and at country level while the later was conducted using primary data sources and at district level. Moreover, the level of the health seeking behavior among mothers who had ANC follow-up in the two studies might have affected the prevalence of the home delivery.

On the other hand, the prevalence of home delivery among women who had ANC follow up in this study was higher than the prevalence of home delivery in the studies done in Debremarkos (25.3%) (13) and Wolayita zone (13.5%) (11), Ethiopia. The difference might be due to the fact that this study is done at country level and from secondary data (EMEDHS report) while the later studies were conducted at a district level using primary data sources. Moreover, the difference might also be due to variations in access to health facilities, the level of quality of care and prenatal consultation in the two study settings. This finding implies many women do not have complete ANC visits and this in turn decreases deliveries by skilled birth attendants.

Educational status was significantly associated with home delivery among women on ANC. Women who attained primary, secondary and higher educational levels were less likely to deliver at home compared to women who were uneducated. This finding is in line with the findings of studies in Nepal (21), Uganda (22), and systematic review result in Ethiopia (1). This might be due to the fact that educated women get information on the importance of institutional delivery and this could facilitate behavioral changes that might allow mothers for the acceptance and utilization of maternal health service. This implies that educated women are more alert of the potential risk and complications of home delivery and have a better idea about institutional delivery.

The odds of home delivery were higher among women from rural areas compared to women from urban areas. This is supported by findings from previous studies (23, 24). This might be due to the fact that those with financial capability afford costs of transportation, poor knowledge of institutional delivery services, and less availability of nearby health care services. Moreover, women with richest wealth quintiles afford to have delivery in private clinics which require high payments as compared with the government health institutions whose problems that are related with quality of care are raised.

Regarding geographical region of the country, women from Benishangul, SNNPR, Addis Ababa and Dire Dawa had lower odds of home delivery compared to women from Oromia region. This might be related to the difference in availability of health facilities. In particular, Addis Ababa and Dire Dawa are urban areas where quality of maternal health service is better than other parts of the country and women from these areas are also more informed about the risks of home delivery, and this could reduce the likelihood of delivering at home.

Adequacy of ANC visit was another variable which was significantly associated with home delivery. Women without adequate ANC visit had higher odds of delivering at home. A similar finding was reported in the study in Ethiopia (2, 25). This could be explained by counseling about written birth plan, which is provided at the last ANC visit that might have made women with complete ANC follow up more prone to practice institutional delivery.

In this study women who had first ANC visit lately during the third trimester were less likely to deliver at home compared to those who had first ANC visit during the first trimester of pregnancy. This finding contradicts with a previous finding of a study in Ethiopia (26). Therefore, further studies are needed to resolve the discrepancy.

This study has limitations that should be considered when interpreting the results. Since the present study used secondary data, some important variables like quality of ANC service, media exposure, experience of prolonged labor, planned pregnancy, and maternal knowledge, privacy during ANC and women's decision-making autonomy were not included in the analysis. The result might be prone to recall bias. Additionally, causal association cannot be established between the home delivery and risk factors due to cross-sectional nature of study design.

## Conclusion

Near to one-third of women in Ethiopia who attending at least once ANC visits have delivered their babies at home. Religion, educational status, place of residence, region, wealth index, timing of first antenatal check and adequate ANC visit were significantly associated with home delivery among women who had ANC follow up in the country. Therefore, focused intervention packages need to be implemented at all levels of the health care system in Ethiopia to improve health seeking behaviors of women who have ANC follow up in order for them to have delivery in health care institutions. Health extension workers must also deliver quality health education to hard-to-reach communities as most of women in Ethiopia reside in the rural parts of the country.

## Data availability statement

The original contributions presented in the study are included in the article/supplementary material, further inquiries can be directed to the corresponding author/s.

## Author contributions

Conception of the work, design of the work, acquisition of data, analysis, and interpretation of data were conducted by BT. Data curation, drafting the article, revising it critically for intellectual content, validation, and final approval of the version to be published were done by DE, AW, NB, and MT. All authors read and approved the final manuscript.

## Conflict of interest

The authors declare that the research was conducted in the absence of any commercial or financial relationships that could be construed as a potential conflict of interest.

## Publisher's note

All claims expressed in this article are solely those of the authors and do not necessarily represent those of their affiliated organizations, or those of the publisher, the editors and the reviewers. Any product that may be evaluated in this article, or claim that may be made by its manufacturer, is not guaranteed or endorsed by the publisher.

## Abbreviations

AOR: Adjusted Odds Ratio
ANC: Antenatal Care
CI: Confidence Interval
EA: Enumeration Areas
EMDHS: Ethiopian Mine Demographic Health Survey Demography heath survey
ICC: Intra-cluster Correlation Coefficient
SD: Standard Deviation
SNNPR: Southern Nations, Nationalities and People's Region.
